# Supplementary material for: Inherently confinable split-drive systems in Drosophila
Source: Nat Commun. 2021 Mar 5;12:1480. doi: 10.1038/s41467-021-21771-7 (PMC7935863; doi:10.1038/s41467-021-21771-7)
Supplement: Supplementary file 1 — Supplementary Information [file 41467_2021_21771_MOESM1_ESM.pdf]

Inherently confinable split-drive systems in *Drosophila*

Gerard Terradas[1,2], Anna B. Buchman[1], Jared B. Bennett[3], Isaiah Shriner[1], John M. Marshall[4,5], Omar S. Akbari[1], and Ethan Bier[1,2*]

[1] Section of Cell and Developmental Biology, University of California, San Diego, La Jolla, CA 92093, USA
[2] Tata Institute for Genetics and Society, University of California, San Diego, La Jolla, CA 92093, USA
[3] Biophysics Graduate Group, Division of Biological Sciences, College of Letters and Science, University of California, Berkeley, CA 94720, USA
[4] Division of Biostatistics and Epidemiology - School of Public Health, University of California, Berkeley, CA 94720, USA
[5] Innovative Genomics Institute, Berkeley, CA 94720, USA

* Corresponding author

e-mail: ebier@ucsd.edu

This document contains: Supplementary Figures and Mathematical Information

- Supplementary Figure 1-5
- Supplementary Table 1
-
  Supplementary Methods
- Supplementary Table 2-13
- Supplementary Figure 6-11

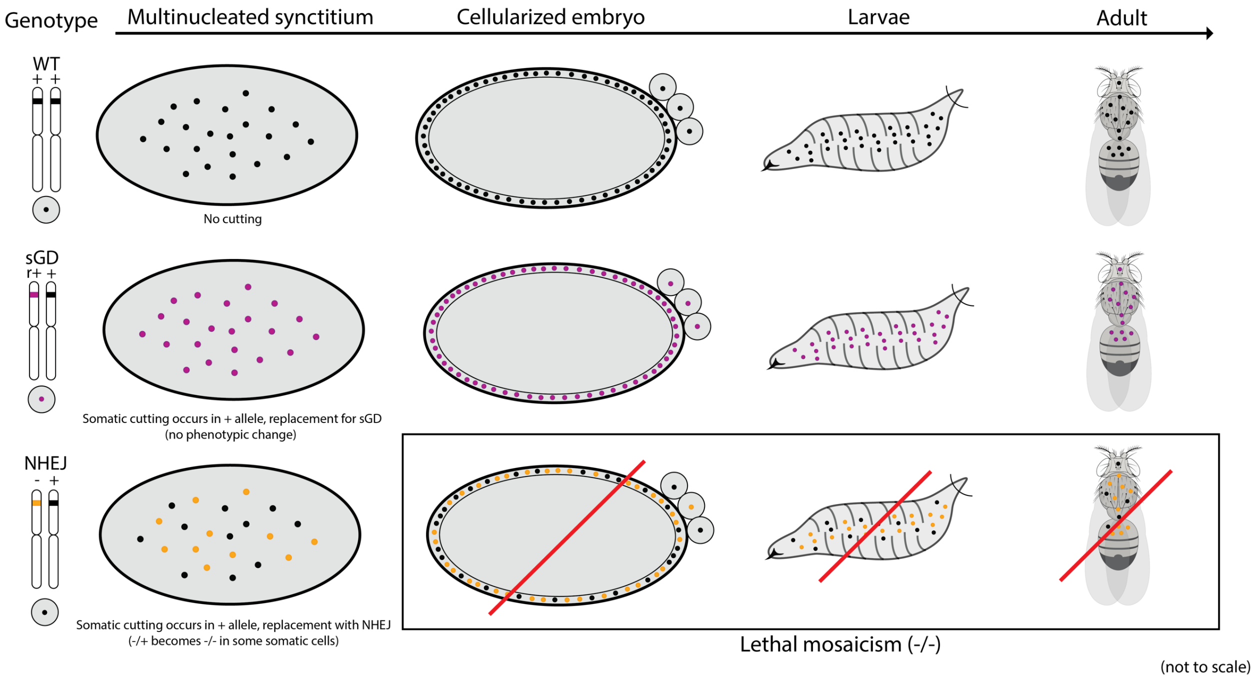

Supplementary Figure 1 - Schematics of the lethal mosaicism effect. Scheme explaining the phenomenon of lethal mosaicism. Each colored dot represents cellular phenotype. Colored dot under the chromosomes represents allelic dominance. The first two rows (WT, + (black)/+ and sGD, r+ (purple)/+) depict successful fly development to adulthood. The bottom row (NHEJ, +/- (orange)) shows the developmental pause following Cas9 cleavage of somatic cells. In the latter, cleavage can somatically occur in the parental WT (+) chromosome and generate mutation through repair by NHEJ. This can lead to the generation of a second indel on the homologous chromosome by formation of a new NHEJ event or by conversion using the already mutated NHEJ allele. Cells that undergo this somatic conversion will present a knockout phenotype (-/-), leading to cell death when the affected gene is deemed essential for survival. Cellular death and the stage at where the knockout phenotype is visible varies depending on the amount of cells affected, time of cleavage and essentiality of the gene.

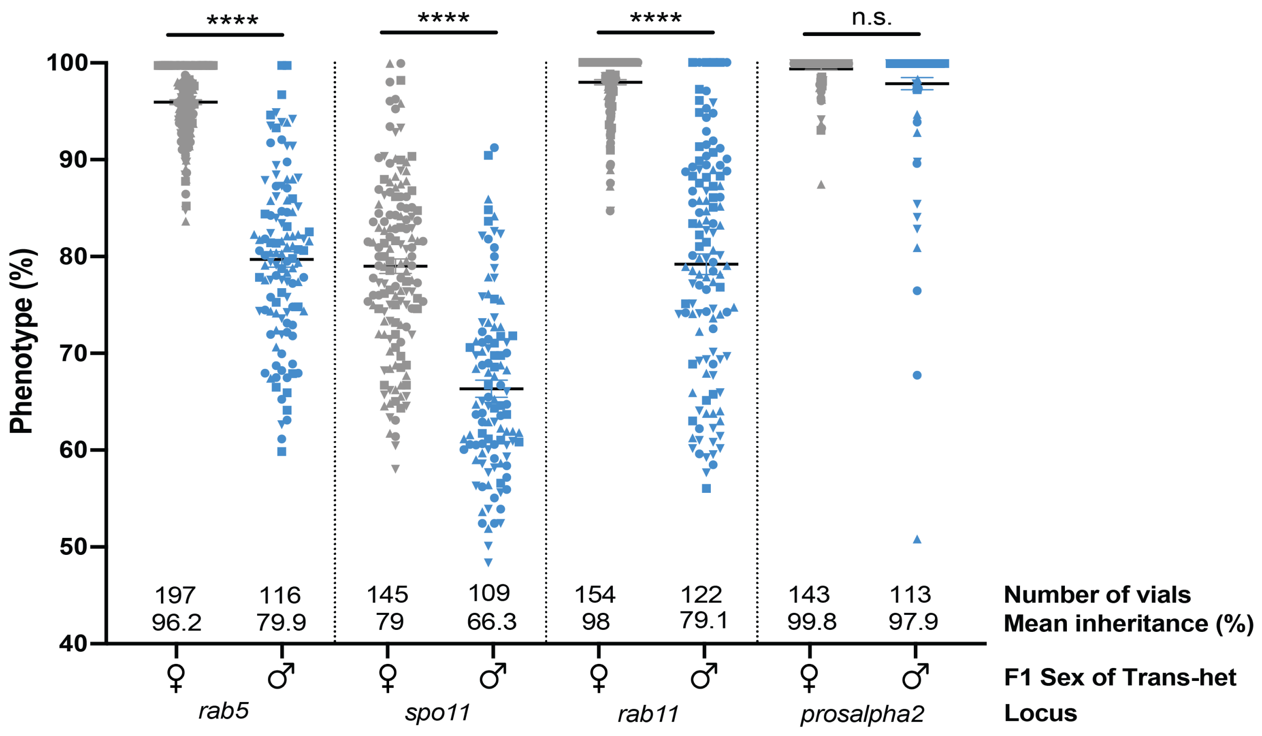

Supplementary Figure 2 – sGD-Cas9 is inherited in Super-Mendelian fashion depending on sex but independent of the Cas9 promoter used. The graph depicts the differences in F_2 inheritance depending on the sex of the F_1 trans-heterozygote where germline chromosomal conversion occurs. For all tested genes except prosalpha2, we observed a 15-20% drop in transgene transmission when conversion occurs in F_1 males (blue) compared to transmission through F_1 females (grey). This graph was obtained by pooling the male or female lineages from Fig 2, without taking into account the Cas9 promoter. Similar to Figure 2, shape of the data points depict promoter used: vCas9 in the III (circle) or X (square) or nCas9 in the III (triangle facing up) or II (down) chromosomes. Error bars represent mean values ± SEM. Stars represent statistical significance (**** p<.0001) for F_1 male-to-female sGD-copying differences (two-sided t-test). Raw phenotypical data is provided as "Supplementary Data 3".

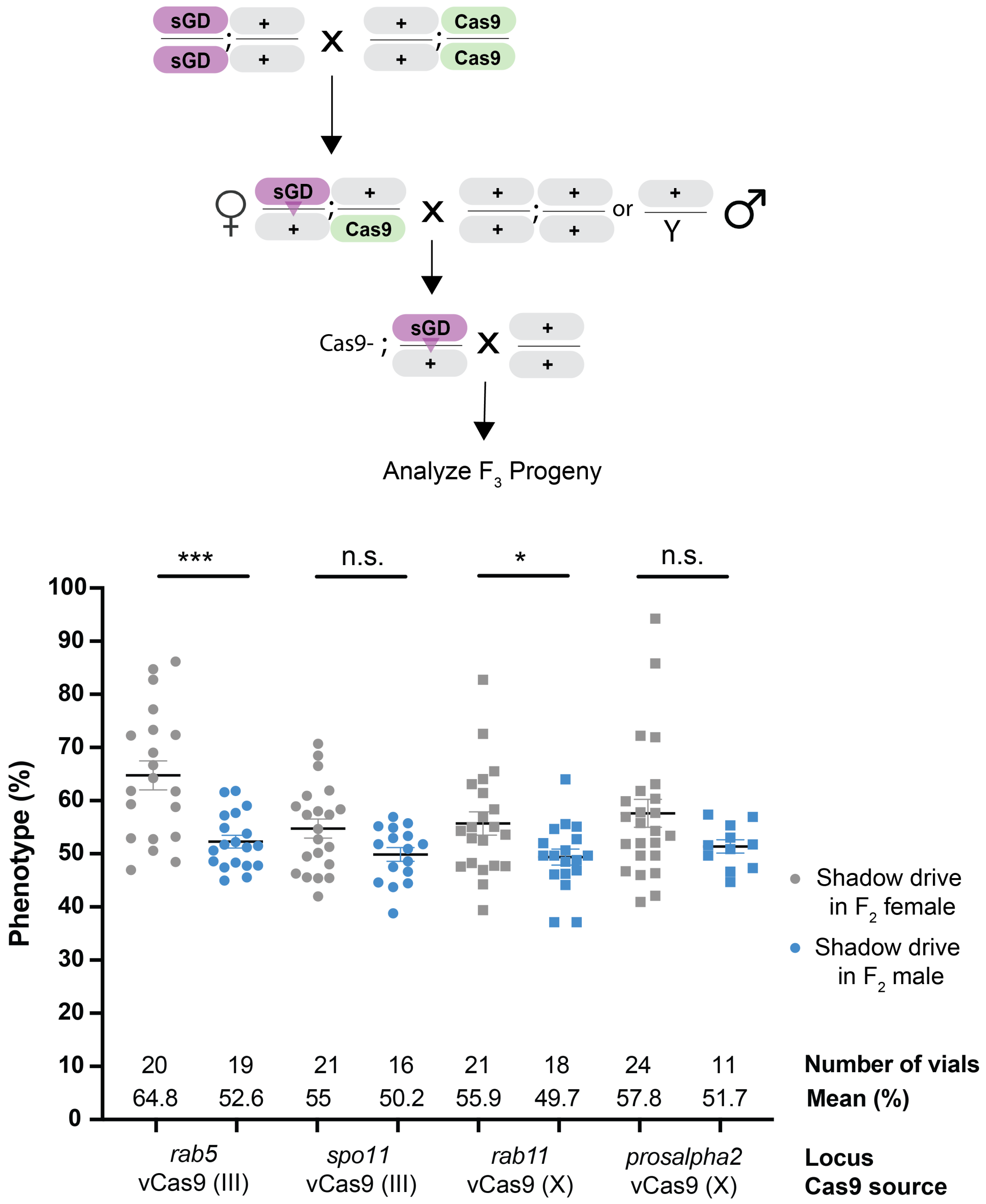

Supplementary Figure 3 - Shadow drive. Maternally-deposited Cas9/gRNA complexes can mediate cleavage of the gRNA target site even in the absence of genetically encoded Cas9 and thus bias transmission rates an extra generation. This phenomenon is referred to as “shadow drive”. To assess the capacity to perform shadow drive for different sGD, we analyzed sGD+/Cas9- F_2 individuals, crossing them to a wild-type individual and scoring for the marker phenotype in F_3 progeny (shown in cross scheme). In grey, sGD transmission from F_2 females; in blue, sGD transmission from F_2 males, which serve as a control since there is no paternal deposition of Cas9 into sperm cells. Circles show data when the specific sGD was combined with vCas9-III while squares depict data using vCas9-X. Error bars represent mean values ± SEM. Raw phenotypical data is provided as “Supplementary Data 3”.

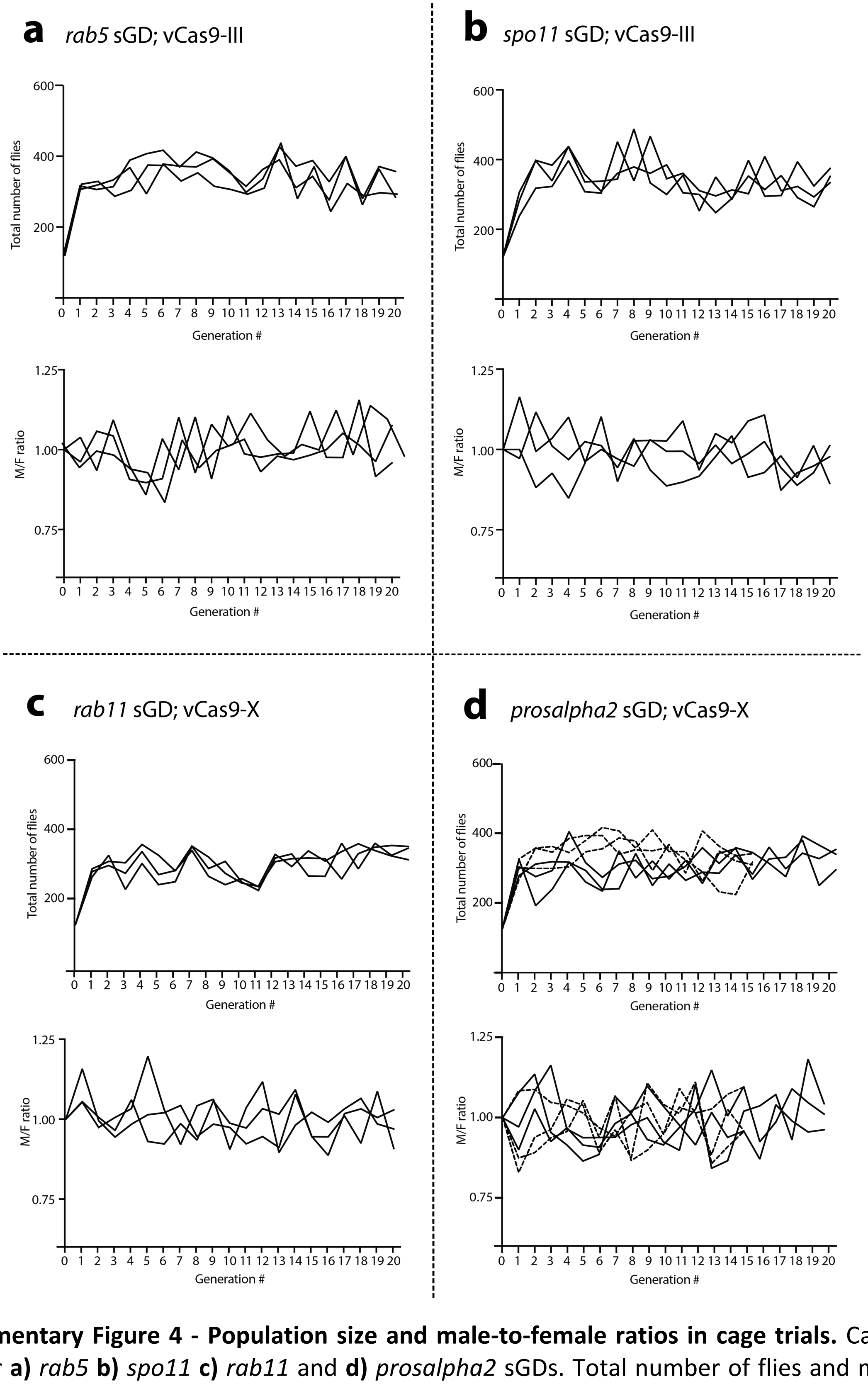

Supplementary Figure 4 - Population size and male-to-female ratios in cage trials. Cage trial data for **a)** *rab5* **b)** *spo11* **c)** *rab11* and **d)** *prosalpha2* sGDs. Total number of flies and male-to-female ratios were monitored at every generation to unveil possible population size reductions or sex preference due to associated fitness costs. In all conditions, we observed random fluctuations of the population size and sex ratios. Raw phenotypical data is provided as "Supplementary Data 2".

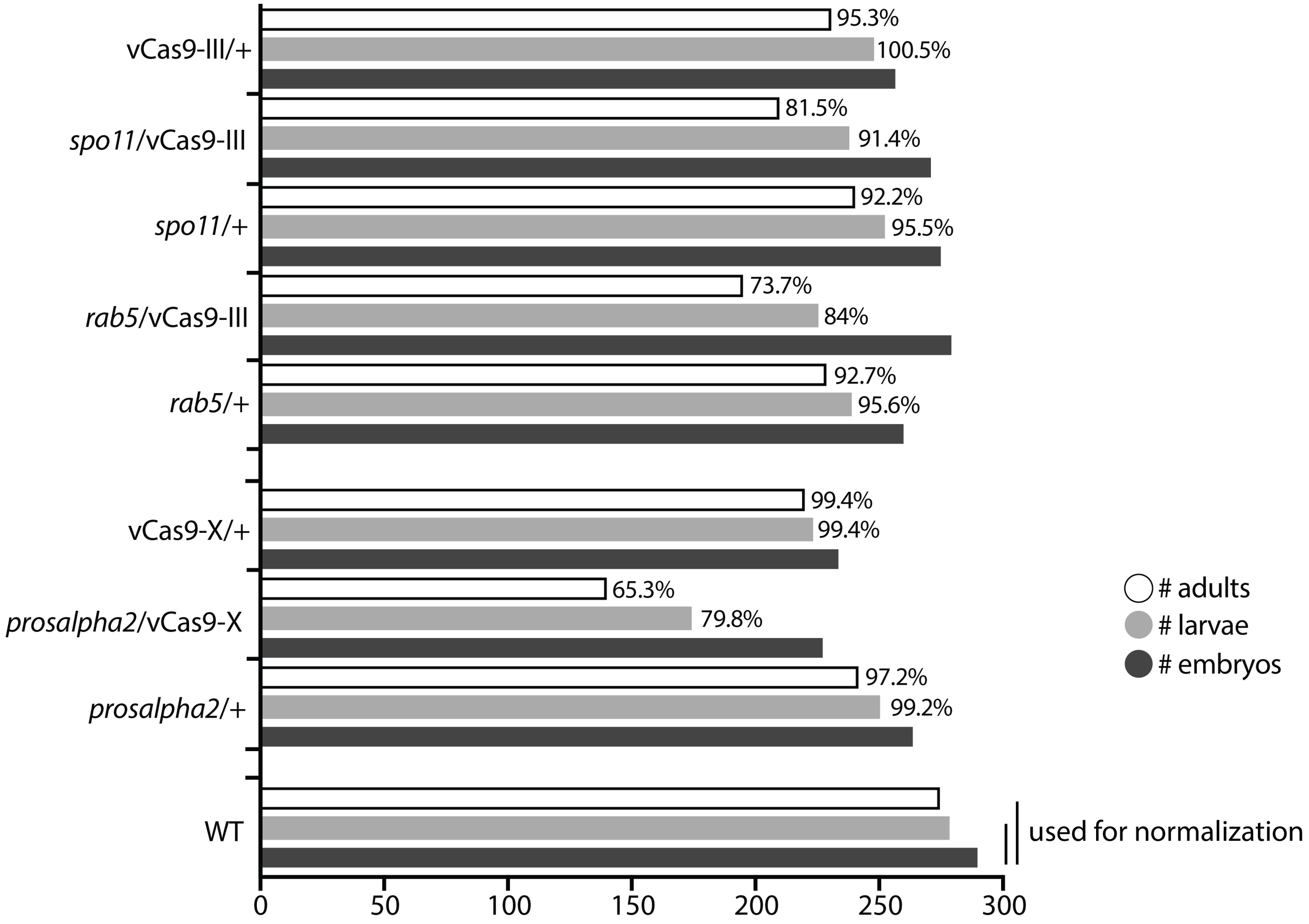

Supplementary Figure 5 - Egg-laying and hatchability assays. To account for potential fecundity-associated costs in transgenic flies, we performed a series of egg-deposition and viability experiments. The graph shows number of laid embryos (black) number of those that hatch (grey), and how many reached adulthood (white). Hatching and adult percentages were normalized to WT fly values. Normalization was carried by calculating the egg-to-larvae and egg-to-embryo ratio in WT cages and making it the comparison baseline (100%). Both hatching and development were significantly impaired in transheterozygotes containing both sGD and vCas9, decreasing from >95% to 80%, 84% and 91% for prosalpha2, rab5 and spo11 in the former, and to 65%, 74% and 82% in the latter. Differences demonstrate degrees of embryonic viability and somatic lethal mosaicism.

Supplementary Table 1 – Primers (5' to 3') used for different experimental assays in the paper.

**For construct homology arms**		
rab5 sGD	1069 C1	GGTTAATTCGAGCTCGCCCGGGGTCCTAGG**CAGTTGCCATCTCAGAGGAGG**
	1069 C4	GGTGGGGAGGCCACCGAGTATGGGCGCGCC**GTGGAAAATAGTTCAACGC**
spo11 sGD	1069 E1	GGTTAATTCGAGCTCGCCCGGGGTCCTAGG**GCGCTCTCTTCATTTGCAACTAG**
	1069 E4	GGGGAGGCCACCGAGTATGGGCGCGCC**GGAAAGCTGATTACGCGAAC**
rab11 sGD	1069 D1	GGTTAATTCGAGCTCGCCCGGGGTCCTAGG**GCCAAAATGTTGACATCATAGTTGTCACT**
	1069 D4	GGTGGGGAGGCCACCGAGTATGGGCGCGCC**GCGTCTTCGGATGCACTTTC**
prosalpha2 sGD	GT143	**GGTCACTTTATCGAGTCCACGTG**
	GT144	**GAGGCCATCGGAGCGAAGTG**
**To check insertions (outside HAs)**		
rab5 sGD	GT176	**CACACTCAGCAGCAATCTTAGCAG**
	GT178	**CGGATCTTTGATGTTTGTGGAACG**
spo11 sGD	GT177	**CTCTGAGCGAGAGAGCTAATAGC**
	GT179	**CCACTCAATACGAAACACTAGAAGCAG**
rab11 sGD	GT201	**GTTTGCAACGCGCAGCATAG**
	GT202	**GCGATGGATGACGAAGGAGTG**
prosalpha2 sGD	GT199	**CTACACTTACTTGAGCACGCTG**
	GT200	**CGCCACACCGTAATCTGTTC**
**For sequencing NHEJ (single cross)**		
rab5 sGD	GT135	**GTGCGAAGACCTGGGTCAAG**
spo11 sGD	GT133	**CCTCGAGTTGCTGAGCAACTTG**
rab11 sGD	GT137	**CGTTGAAACGGCATTCCAGAAC**
prosalpha2 sGD	GT207	**GTCACACTGCCGCCCAATTG**
**For sequencing NHEJ (cage trials)**		
*rab5*_seq	F	ACACTCTTTCCCTACACGACGCTCTTCCGATCT**GAGGAGAACGGGCTGCTGTTC**
	R	GACTGGAGTTCAGACGTGTGCTCTTCCGATCT**CTGCGAGAGCCGGCTTTAGG**
*spo11*_seq	F	ACACTCTTTCCCTACACGACGCTCTTCCGATCT**GCGTGCTGATTTACATGCTCTC**
	R	GACTGGAGTTCAGACGTGTGCTCTTCCGATCT**GGCAATGTCAGAGGTCCACC**
*rab11*_seq	F	ACACTCTTTCCCTACACGACGCTCTTCCGATCT**CGCATTGTGTCGCAGAAACAG**
	R	GACTGGAGTTCAGACGTGTGCTCTTCCGATCT**CATCGGGTGTACGCTGCTC**
*prosalpha2*_seq	F	ACACTCTTTCCCTACACGACGCTCTTCCGATCT**TGGCTACCGAACGATACAGC**
	R	GACTGGAGTTCAGACGTGTGCTCTTCCGATCT**CAATGACGACGCCGTTGGAAG**

**Cloning strategies**		
prosalpha2 sGD	Cloning by RE	
rab5 sGD	1069 C1	GGTTAATTCGAGCTCGCCCGGGGTCCTAGGCAGTTGCCATCTCAGAGGAGG
	1069 C2	GCTCGAATTTTAATTAACGTCGGTCGATTTGTTTCGG
	1069 C3	CAATGTATCTTAAAGCTTCCGCGGAACAACTGCTGCAAGTGATGTCC
	1069 C4	GGTGGGGAGGCCACCGAGTATGGGCGCGCCGTGGAAAATAGTTCAACGC
	1069 C5	CATCACTTGCAGCAGTTGTTCCGCGGAAGCTTTAAGATACATTGATGAGTTTGGAC
	1069 C6	CGACCGACGTTAATTAAAATTCGAGCTCGCCCGGGGATC
	1069 C7	GGAACCGAAACAAATCGACCGACGAATAATTGTTGTAAATAAAATGAATCGTTTTTAAAATAACAAATCAATTG
	1069 C10	CTAAAACACGAACAACTGCTGCAAGTGGACGTTAAATTGAAAATAGGTCTATATATACG
rab11 sGD	1069 D1	GGTTAATTCGAGCTCGCCCGGGGTCCTAGGGCCAAAATGTTGACATCATAGTTGTCACTTGTGC
	1069 D2	CTCGAATTTTAATTAACGGCGGTGACAGTCGGCTTTAC
	1069 D3	GTATCTTAAAGCTTCCGCGGATGTGCGCAAACAGTGCTGTC
	1069 D4	GGTGGGGAGGCCACCGAGTATGGGCGCGCCGCGTCTTCGGATGCACTTTC
	1069 D5	CTGTTTGCGCACATCCGCGGAAGCTTTAAGATACATTGATGAGTTTGGAC
	1069 D6	CTGTCACCGCCGTTAATTAAAATTCGAGCTCGCCCGGGGATC
	1069 D7	GTAAAGCCGACTGTCACCGCCGTTAATTAAACGTCCGTAAGCAATGTTGCCAATAAAATGAATCGTTTTTAAAATAACAAATCAATTG
	1069 D10	ACGAATTCGAGCTCGCCCGGGACGTTAAATTGAAAATAGGTCTATATATACG
spo11 sGD	1069 E1	GGTTAATTCGAGCTCGCCCGGGGTCCTAGGGCGCTCTCTTCATTTGCAACTAG
	1069 E2	GGCGAGCTCGAATTCCACGGACGGTGAAACTTCC
	1069 E3	CAATGTATCTTAAAGCTTCCGCGGACTATACTATGACAATCCTCTGCTAGTC
	1069 E4	GGGGAGGCCACCGAGTATGGGCGCGCCGGAAAGCTGATTACGCGAAC
	1069 E5	GTCATAGTATAGTCCGCGGAAGCTTTAAGATACATTGATGAGTTTGGAC
	1069 E6	ACCGTCCGTGGAATTCGAGCTCGCCCGGGGATC
	1069 E10	CTGGACTATACTATGACAATCGACGTTAAATTGAAAATAGGTCTATATATACG
	1069 E11	CGTCGATTGTCATAGTATAGTCCAGTTTTAGAGCTAGAAATAGCAAGTTAAAATAAGG
	1069 E12	GCGAGCTCGAATTACAAAAGCTGGAGCTCCTGC
	1069 E13	GTAATACAGTCCACGGACGGTGAAACTTCC
	1069 E14	CTCCAGCTTTTGTAATTCGAGCTCGCCCGGGGATC
Shared	1069_act88_F	GTCTTTCGCCCGCCGCGAAA
	1069_act88_R	GACCGGCTGCCAGATTTTCAACGTTCAATCG
	1069_GFPhack	TTGAAAATCTGGCAGCCGGTCACTGCACGCCGTAGGTCAGG
	1069_Opie2hack	CATACTCGGTGGCCTCCCCAC
	1069_tomatoHack	TTTCGCGGCGGGCGAAAGACACCAGTCCTCCGTCTTCGAAATTC
	1069A_hack	GTGGGGAGGCCACCGAGTATGCATTGGCGGCCGTATCTGGC
	1069B_hack	GTGGGGAGGCCACCGAGTATGCATTTTTGCTTTCCTCGAAACAATATTTCG

	1069C_hack	GTGGGGAGGCCACCGAGTATGAACAACTGCTGCAAGTGATGTCC
	1069D_hack	GTGGGGAGGCCACCGAGTATGATGTGCGCAAACAGTGCTGTC
	1069E_hack	GTGGGGAGGCCACCGAGTATGACTATACTATGACAATCCTCTGCTAGTCCG
	1069 CD8	GGTGAGCAAAAAACCGTTAACTCGAATCGCTATC
	1069 CDE9	GATTCGAGTTAACGGTTTTTTGCTCACCTGTGATTGCTC
	1069 CDE12	GATCCCCGGGCGAGCTCGAATTTTAATTAAACAAAAGCTGGAGCTCCTGC

SUPPLEMENTARY METHODS

Mathematical Model Fitting:

We model the population dynamics of split-drive in one of three ways, corresponding to the three design choices implemented. Each of these manifests in how genotypes are assigned and impacts how phenotypes are mapped to genotypes, but does not impact the overall methodology. The overall model fitting procedure is therefore consistent, and we acknowledge implementation differences when they occur.

The basic split-drive is a double-locus system, with the Cas9 at the first locus and gRNAs at the second. Under laboratory cage conditions, we assume discrete generations and a randomly mixing population. Model fitting was carried out using a likelihood-based Markov chain Monte Carlo (MCMC) algorithm to sample from parameter space and explore parameter distributions. Here, we describe the phenotype-genotype mapping for the system, the likelihood calculation, and specific parameter implementations that differ between each construct.

A) The first split-drive design is implemented on two autosomes; Cas9 on chromosome 3, labeled with GFP, and the gRNAs located on chromosome 2, labeled with tdTomato. To handle the effects of shadow drive (in which Cas9 is deposited in the embryo of a mother who doesn't have the Cas9 allele, but whose own mother does), we implement three alleles at the Cas9 locus: W, the wild-type allele, C, the Cas9 (marked with GFP) allele, and S, the shadow drive allele (for females who have the W allele, but whose mothers have the C allele). At the gRNA locus, as homing occurs here, we implement four alleles: W, the wild-type allele, G, the gRNA (marked with tdTomato) allele, and R/B, which are functional/non-functional resistance alleles. These alleles, and their corresponding marker phenotypes, are summarized in Table S1. As flies are diploid, this allele scheme implies five unique and viable genotypes at locus 1 and ten genotypes at locus 2. Since the two loci assort independently, this means we have 50 possible genotypes, which based on the fluorescent labels, fall into four phenotype categories. The resulting genotype-phenotype mappings are summarized in Table S4.

B) The second split-drive design implements both constructs on chromosome 3, close enough that cross-over is negligible. At locus 1, the Cas9 locus, there are the same three possible alleles; W, wild-type, C, Cas9 (marked with GFP), and S, the shadow drive allele. At locus 2 are the gRNAs, with the same four possible alleles: W, wild-type, G, gRNA (marked with tdTomato), and R/B, functional/non-functional resistance alleles. These alleles are summarized in Table S2, which is very similar to Table S1. Each chromosome assorts independently; but the two loci are now linked, so there are 12 possible allele combinations on each chromosome, generating 68 unique possible genotypes. These resulting genotype-phenotype mappings are summarized in Table S5.

C) The third and final split-drive design is an X-Linked arrangement. There are three possible versions of the X chromosome; X, the wild-type, C, Cas9 (marked with GFP), and S, the shadow drive allele. Additionally, we inserted a Y allele to designate males, such that all males are some combination of Y and X, C or S, while females are any diploid combination of X, C or S. The second locus is the same for males and females, and has four possible alleles; W, wild-type, G, gRNA (marked with tdTomato), and R/B, functional/non-functional resistance alleles. These alleles are summarized in Table S3. The X/Y combinations assort independently, and sex is determined accordingly, while the second locus assorts completely independently. Thus, there are 50 unique female genotypes and 30 unique male genotypes, for a total of 80 distinct genotypes in the simulation. These resulting genotype-phenotype mappings are summarized in Table S6.

Given the large number of possible mating pairs, it is not feasible to show the complete equations for the next generation genotype frequencies, so we instead define a set of rules that describe offspring genotype frequencies for all mating classes, and explain their application using examples for each mating class. While all crosses accept sex-specific parameters, the general equations are agnostic to sex.

The simplest inheritance scenarios are those where there is no gene drive involved. As this is a split-drive system, both the Cas9 and gRNA alleles must be present for homing to occur. Individuals without at least one copy of each of the Cas9 and gRNA alleles therefore abide by

Mendelian inheritance rules. The only exception to this is when shadow drive occurs, a phenomenon whereby female offspring of mothers with Cas9 receive a certain amount of Cas9 protein deposited during oogenesis, but no Cas9 allele. In the presence of this deposition, along with a copy of the gRNAs, a limited amount of homing is possible. Due to the rareness of this event and the size of the cage trials, it was not possible to estimate the ratio of accurate homology-directed repair (HDR) versus resistance allele formation as a result of this phenomenon. Additionally, single-pair crosses indicate a high frequency of uncleaved wild-type alleles under these conditions (Main Supplementary, Supp Fig 4), indicating very high HDR rates but potentially minimal cutting rates. Therefore, we estimated a shadow drive-mediated cleavage rate, c^S, such that the remainder of alleles, $1 - c^S$, remain wild-type. We assume that all alleles cleaved through the shadow drive mechanism are repaired through accurate HDR. This parameter applies to females only, as experiments indicate no shadow drive effect in males (Main Supplementary, Figure S4), potentially due to the reduced protein load within sperm.

When at least one of each of the Cas9 and gRNA alleles are present in an individual, the pieces combine to promote Cas9-mediated cleavage and repair. We do not fit copy-number dependent parameters in this analysis, therefore having one Cas9 allele provides the same cleavage rate as two copies. In the presence of both Cas9 and gRNA alleles, a fraction, c_F^H (c_M^H), of wild-type alleles are cleaved in females (and males), while the remaining $1 - c_F^H$ $(1 - c_M^H)$remain wild-type in females (and males). Given cleavage, accurate HDR occurs at a rate p_F^{HDR} (p_M^{HDR}), while the remaining $1 - p_F^{HDR}(p_M^{HDR})$ alleles undergoing some form of non-homologous end-joining (NHEJ). For alleles that are cleaved, but do not undergo HDR, a fraction p_F^{NHEJ} (p_M^{NHEJ}) are repaired in-frame, creating functional alleles, while the remaining $1 - p_F^{NHEJ}$ $(1 - p_M^{NHEJ})$ are repaired out-of-frame, generating nonsense mutations and creating non-functional alleles. Parameters for each drive design are listed in Tables S7-S12, including a short explanation of each parameter, and maximum likelihood parameter estimates from model fitting.

In addition to parameters determining inheritance bias, the gene drive system is associated with fitness costs through multiple mechanisms: i) possession of a functional Cas9 element in conjunction with a viable gRNA construct, and ii) NHEJ events at the gRNA locus. We

disregard assortative mating issues here because all experiments were performed in a *white*-background. It was hypothesized that costs from Cas9 or gRNA alone were insignificant compared to costs from Cas9 and gRNA co-occurring. Early model fits supported this hypothesis, and so subsequent fits were performed estimating the combined cost of the loaded Cas9/gRNA complex. The possession of this active complex was modeled as leading to reduced fecundity (in females) and reduced mating competitiveness (in males), denoted in both cases by the fitness cost, $s_{c/g}$. This cost was applied in a copy-dependent fashion, so individuals homozygous for the Cas9 and gRNA alleles experience a fitness reduction of $2\ s_{c/g}$.

Fitness reductions due to loss-of-function (LOF) mutations ("B" alleles, non-functional NHEJ events) manifest differently from Cas9/gRNA costs. Spo11 impacts female fecundity, and so females homozygous for LOF alleles lay no eggs, while males are unaffected. Rab5 and Rab11 impact cell signaling and prosalpha2 impacts protein degradation, both of which affect egg maturation, and are implemented as reductions in egg viability (again, males are unaffected). For all constructs, we fitted a fitness cost, s_{NHEJ}, associated with "heterozygous" LOF allele carriers. We quote "heterozygous" here because heterozygotes are viable and fit (the LOF mutations are recessive in fitness impact), so what this parameter actually estimates is the fitness consequence of a degree of somatic mosaicism. The extent and bodily location of somatic mosaicism greatly impacts the fecundity reduction due to the LOF mutations, and is itself dependent on the genomic location of each construct.

Fitness is defined relative to wild-type organisms. As we apply several fitness costs additively, we have to ensure that this results in a cumulative fitness cost between 0 and 1. We achieved this by truncating the total cost for an individual at 100%. Thus, if an individual has one Cas9 allele (in addition to at least one gRNA allele) and one LOF allele, their fitness cost is $min((s_{c/g} + s_{NHEJ}),1)$. This is intuitive biologically, as having two costly alleles implies that the organism experiences the fitness impact of both; however, it ignores any synergistic or nonlinear effects resulting from combining several costs in one organism.

Considering these inheritance biases and fitness costs, we calculate the expected genotype frequencies in each generation, denoted p_k^x for genotype x in generation k. Genotype frequencies are normalized at each generation to ensure they sum to 1.

The likelihood of the population cage data was calculated by assuming a multinomial distribution of individuals having each sex and marker phenotype, and by using the model predictions to generate expected proportions for each set of parameter values. I.e., by calculating the log likelihood,

$$logL(\theta) \propto \sum_{i=1}^{3} \sum_{k=1}^{n_i} \sum_{P \in \{P_F, P_M\}} N_{i,k}^{P} \cdot log(p_k^P(\theta))$$

Here, the log-likelihood is a summation over the three replicates for each system, *i*, over each generation, *k*, and over each phenotype for females, P_F, and males, P_M, denoted collectively by P. $N_{i,k}^{P}$ denotes the number of individuals at generation *k* in experiment *i* having phenotype P. Possible phenotypes with corresponding genotype-phenotype mappings are given in Tables S4-S6. The *i*th experiment is run for n_i generations, and expected phenotype frequencies, $p_k^P(\theta)$, at generation *k* are dependent on the model parameters, $\theta = \{c_F^H, p_F^{HDR}, p_F^{NHEJ}, c_M^H, p_M^{HDR}, p_M^{NHEJ}, c^S, s_{c/g}, s_{NHEJ}\}$ (see Tables S7-S12 for parameter definitions and estimates for each construct).

Experiments were seeded with a mixture of wild-type and heterozygous gene drive flies. This mixture was dependent on the gene drive design:

A) The two autosome split-drive designs began with 120 flies, equally split between sexes, with 75% (90 count) wild-type and 25% (30 count) heterozygous for the Cas9 and gRNA elements. this corresponds to 90 “WWWW” and 30 “CWGW” flies, using genotypes from Table S4.
B) The single autosome design began with 120 flies, equally split between sexes, with 75% wild-type (90 count) and 25% (30 count) trans-heterozgyous for Cas9 and gRNA. This was done using 90 “WWWW” and 30 “CWWG” flies, per the genotype designations in Table S5.
C) The X-Linked arrangement was initialized in two ways. This was done because prosalpha2 showed a significantly higher fitness cost than the other drives tested, so another cage

trial was performed to explore the cause of that cost. All designs were seeded with 120 flies, split equally between sexes.

a. Two of the X-linked designs began 75% wild-type (90 count) and 25% (30 count) heterozygous for the drive. In males, this implies 45 flies “XYWW” and 15 flies “CYGW,” and in females it implies 45 flies “XXWW” and 15 flies “CXGW”, per the genotypes in Table S6.
b. The extra prosalpha2 trial was done in a Cas9 background. It began 75% Cas9:Cas9 with no gRNA (90 count) and 25% (30 count) Cas9:Cas9 and heterozygous for the gRNA allele. Females began completely Cas9 positive, 60 flies with the “CCWW” genotype. Males began with 30 “CYWW” “wild-type” and 30 “CYGW” drive positive, according to the genotypes in Table S6.

Models were fitted using a Differential-Evolution MCMC procedure with snooker updating from the R package BayesianTools. The fitting procedure was run five times for 450,000 iterations per repetition. The estimated parameters, along with some statistics about each parameter, are included in Tables S7-S12 (each table corresponds to one cage trial). Each fit is visualized with the corresponding cage trial data in Figures S1-S6.

Stochastic realizations, using a discrete-generation adaptation of MGDrivE, were performed using the fitted parameters described above. Here, mating follows a multinomial distribution over male genotypes, accounting for the male fitness costs described above. Egg-laying follows a multinomial distribution over offspring genotype, determined by maternal and paternal genotypes and the specific split-drive being simulated. Number of offspring is Poisson-distributed, dependent on genotype-specific female fecundity, and sex distribution of offspring follows a binomial distribution, assuming equal probability. Adults in each generation are sampled equally for each sex, with an expected population size equal to the average size of each experimental generation, following a multivariate hypergeometric distribution. The trajectories from these simulations are depicted in Figure 5.

All simulations were performed, analyzed and plotted in R (R Core Team, https://www.r-project.org), with code/data available upon request.

Locus 1 (Chromosome III)			Locus 2 (Chromosome II)		
**Allele**	**Description**	**Fluorescent label**	**Allele**	**Description**	**Fluorescent label**
W	Wild-type	Null	W	Wild-type	Null
C	Cas9	GFP	G	gRNAs	tdTomato
S	Shadow drive	Null	R	Functional NHEJ	Null
			B	Non-functional NHEJ	Null

Supplementary Table 2 - Autosomal split-drive allele descriptions and fluorescent labels. Locus 1, located on chromosome 3, carries the Cas9 (denoted C, labeled with GFP) allele. When there is no Cas9 present, that locus can take either the wild-type (W) designation, or in special circumstances, the shadow drive (S) designation, neither of which have a fluorescent label. The S "allele" denotes the case where females are wild-type at the Cas9 locus, but their mothers have the C allele, which they inherit through protein deposition from their mother. As the Cas9 locus is static, it is always inherited in a Mendelian fashion, so resistance alleles are not relevant. Locus 2, located on chromosome II, contains the gRNA (G, labeled with tdTomato) locus. When not present, the wild-type (W) allele and NHEJ alleles all appear without fluorescent labels. NHEJ alleles fall into one of two categories: functional (denoted R for "resistant") alleles, and non-functional (denoted B for "broken") alleles.

Locus 1 (Chromosome III)			Locus 2 (Chromosome III)		
**Allele**	**Description**	**Fluorescent label**	**Allele**	**Description**	**Fluorescent label**
W	Wild-type	Null	W	Wild-type	Null
C	Cas9	GFP	G	gRNAs	tdTomato
S	Shadow drive	Null	R	Functional NHEJ	Null
			B	Non-functional NHEJ	Null

Supplementary Table 3 - Autosomal "linked" split-drive allele descriptions and fluorescent labels. Locus 1, located on chromosome III, carries the Cas9 (denoted C, labeled with GFP) allele. When there is no Cas9 present, that locus can take either the wild-type (W) designation, or in special circumstances, the shadow drive (S) designation, neither of which have a fluorescent label. The S "allele" denotes the case where females are wild-type at the Cas9 locus, but their mothers have the C allele, which they inherit through protein deposition from their mother. As the Cas9 locus is static, it is always inherited in a Mendelian fashion, so resistance alleles are not relevant. Locus 2, located on chromosome III, is close enough to Locus 1 that cross-over events are negligible, contains the gRNA (G, labeled with tdTomato) locus. When not present, the wild-type (W) allele and NHEJ alleles all appear without fluorescent labels. NHEJ alleles fall into one of two categories: functional (denoted R for "resistant") alleles, and non-functional (denoted B for "broken") alleles.

Locus 1 (X chromosome)			Locus 2 (Chromosome III)		
**Allele**	**Description**	**Fluorescent label**	**Allele**	**Description**	**Fluorescent label**
X	Wild-type X chromosome	Null	W	Wild-type	Null
Y	Wild-type not-X chromosome	Null	G	gRNAs	tdTomato
C	Cas9	GFP	R	Functional NHEJ	Null
S	Shadow drive	Null	B	Non-functional NHEJ	Null

Supplementary Table 4 - X-linked split-drive allele descriptions and fluorescent labels. Locus 1, located on the X chromosome, carries the Cas9 (denoted C, labeled with GFP) allele. When there is no Cas9 present, that locus can take either the wild-type (X) designation, or in special circumstances, the shadow drive (S) designation, neither of which have a fluorescent label. The S "allele" denotes the case where females are wild-type at the Cas9 locus, but their mothers have the C allele, which they inherit through protein deposition from their mother. As the Cas9 locus is static, it is always inherited in a Mendelian fashion, so resistance alleles are not relevant. Additionally, even though male flies are XO, we use "Y" to designate the lack of X chromosome, for ease of programming. All males in the simulation carry a Y chromosome, paired with an X, C or S chromosome. Locus 2, located on chromosome III, contains the gRNA (G, labeled with tdTomato) locus. When not present, the wild-type (W) allele and NHEJ alleles all appear without fluorescent labels. NHEJ alleles fall into one of two categories: functional (denoted R for "resistant") alleles, and non-functional (denoted B for "broken") alleles.

Observed phenotype	Possible genotype
White	WWWW, WWRW, WWBW, WWRR, WWBR, WWBB, SWWW, SWRW, SWBW, SWRR, SWBR, SWBB
Green (GFP)	CWWW, CWRW, CWBW, CWRR, CWBR, CWBB, CCWW, CCRW, CCBW, CCRR, CCBR, CCBB, CSWW, CSRW, CSBW, CSRR, CSBR, CSBB
Red (tdTom)	WWGW, WWGG, WWGR, WWBG, SWGW, SWGG, SWGR, SWBG
Both (GFP/tdTom)	CWGW, CWGG, CWGR, CWBG, CCGW, CCGG, CCGR, CCBG, CSGW, CSGG, CSGR, CSBG

Supplementary Table 5 - Autosomal split-drive phenotype-to-genotype mappings. As there are four fluorescent labels (GFP, tdTomato, and none, see Table S1), there are four possible phenotypes. These phenotypes provide some indication of the genotype of an individual, as each genotype generates a specific phenotype. From that, we can classify all genotypes as one of four phenotypes observed in the experiments.

For each genotype, the first two letters denote locus 1 (W, C, S), and the latter two letters denote locus 2 (W, G, R, B). As the loci are on different chromosomes, they segregate independently.

Observed phenotype	Possible genotype
White	SBWB, SBWR, SBWW, WBWB, SRWB, WBWR, SWWB, WBWW, SRWR, SRWW, WRWR, SWWR, WRWW, SWWW, WWWW
Green (GFP)	CBCB, CBSB, CBWB, CBCR, CBSR, CBWR, CBCW, CBSW, CBWW, CRSB, CWSB, CRWB, CWWB, CRCR, CRSR, CRWR, CRCW, CRSW, CRWW, CWSR, CWWR, CWCW, CWSW, CWWW
Red (tdTom)	SBWG, SGWB, WBWG, SGWG, SGWR, SGWW, WGWG, SRWG, WGWR, SWWG, WGWW
Both (GFP/tdTom)	CBCG, CBSG, CBWG, CGSB, CGWB, CGCG, CGSG, CGWG, CGCR, CGSR, CGWR, CGCW, CGSW, CGWW, CRSG, CWSG, CRWG, CWWG

Supplementary Table 6 - "Linked" autosomal split-drive phenotype-to-genotype mappings. As there are three fluorescent labels (GFP, tdTomato, and none, see Table S2), there are four possible phenotypes. These phenotypes provide some indication of the genotype of an individual, as each genotype generates a specific phenotype. From that, we can classify all genotypes as one of four phenotypes observed in the experiments. For each genotype, the first two letters denote one allele, the latter two letters denote the other allele. Within each pair of letters, the first letter is locus 1 (W, C, S), and the second letter is locus 2 (W, G, R, B). This slightly more complicated design was chosen to maintain the haplotype phasing for each locus.

Observed phenotype	Possible genotype	
	Female	Male
White	XXWW, XXRW, XXBW, XXRR, XXBR, XXBB, SXWW, SXRW, SXBW, SXRR, SXBR, SXBB	XYWW, XYRW, XYBW, XYRR, XYBR, XYBB, SYWW, SYRW, SYBW, SYRR, SYBR, SYBB
Green (GFP)	CXWW, CXRW, CXBW, CXRR, CXBR, CXBB, CCWW, CCRW, CCBW, CCRR, CCBR, CCBB, CSWW, CSRW, CSBW, CSRR, CSBR, CSBB	CYWW, CYRW, CYBW, CYRR, CYBR, CYBB
Red (tdTom)	XXGW, XXGG, XXGR, XXBG, SXGW, SXGG, SXGR, SXBG	XYGW, XYGG, XYGR, XYBG, SYGW, SYGG, SYGR, SYBG
Both (GFP/ tdTom)	CXGW, CXGG, CXGR, CXBG, CCGW, CCGG, CCGR, CCBG, CSGW, CSGG, CSGR, CSBG	CYGW, CYGG, CYGR, CYBG

Supplementary Table 7 - X-linked split-drive phenotype-to-genotype mappings. As there are three fluorescent labels (GFP, tdTomato, and none, see Table S3), there are four possible phenotypes. These phenotypes provide some indication of the genotype of an individual, as each genotype generates a specific phenotype. From that, we can classify all genotypes as one of four phenotypes observed in the experiments. For each genotype, the first two letters denote the X chromosome, and the latter two letters denote chromosome III. Females are X, C, or S in the first 2 letters, while males are X, C, or S paired with a Y. As chromosome III is an autosome, females and males have the same combinations of alleles at that locus.

Parameters		Differential evolution MCMC parameter estimates			
**Label**	**Description**	**M.A.P.**	**2.5% quantile**	**Median**	**97.5% quantile**
c_F^H	Female cleavage Rate	1.000	0.979	0.997	1.000
p_F^{HDR}	Female HDR rate	0.99	0.882	0.98	0.999
p_F^{NHEJ}	Female functional NHEJ rate	0.362	0.039	0.397	0.497
c_M^H	Male cleavage rate	0.999	0.979	0.996	1.000
p_M^{HDR}	Male HDR rate	0.678	0.502	0.646	0.734
p_M^{NHEJ}	Male functional NHEJ rate	0.500	0.462	0.493	0.500
c^S	Shadow drive cleavage rate	0.400	0.370	0.395	0.400
$s_{c/g}$	Fitness cost per Cas9/gRNA allele	0.048	0.010	0.042	0.051
s_{NHEJ}	Fitness cost of single NHEJ allele	0.001	0.000	0.037	0.936

Supplementary Table 8 - Autosomal split-drive parameter estimates - *rab5*. Parameters were estimated by running the log-likelihood (above) through a differential evolution MCMC algorithm. The maximum a posteriori estimate (M.A.P.) is the point that appears most often, and is the best point estimate for our parameters. Additionally, the mean and 95% credible intervals are provided.

Parameters		Differential evolution MCMC parameter estimates			
**Label**	**Description**	**M.A.P.**	**2.5% quantile**	**Median**	**97.5% quantile**
c_F^H	Female cleavage rate	0.999	0.959	0.992	1.000
p_F^{HDR}	Female HDR rate	0.957	0.829	0.960	0.999
p_F^{NHEJ}	Female functional NHEJ rate	0.016	0.002	0.067	0.449
c_M^H	Male cleavage rate	0.999	0.958	0.992	1.000
p_M^{HDR}	Male HDR rate	0.594	0.545	0.620	0.740
p_M^{NHEJ}	Male functional NHEJ rate	0.001	0.000	0.008	0.046
c^S	Shadow drive cleavage rate	0.149	0.128	0.146	0.150
$s_{c/g}$	Fitness cost per Cas9/gRNA allele	0.113	0.106	0.115	0.123
s_{NHEJ}	Fitness cost of single NHEJ allele	0.499	0.469	0.494	0.500

Supplementary Table 9 - Autosomal split-drive parameter estimates - *spo11*. Parameters were estimated by running the log-likelihood (above) through a differential evolution MCMC algorithm. The maximum a posteriori estimate (M.A.P.) is the point that appears most often, and is the best point estimate for our parameters. Additionally, the mean and 95% credible intervals are provided.

Parameters		Differential evolution MCMC parameter estimates			
Label	Description	M.A.P.	2.5% quantile	Median	97.5% quantile
c_F^H	Female cleavage rate	0.995	0.984	0.997	1.000
p_F^{HDR}	Female HDR rate	1.000	0.956	0.996	1.000
p_F^{NHEJ}	Female functional NHEJ rate	0.090	0.004	0.183	0.918
c_M^H	Male cleavage rate	0.999	0.985	0.997	1.000
p_M^{HDR}	Male HDR rate	0.997	0.944	0.995	1.000
p_M^{NHEJ}	Male functional NHEJ rate	0.202	0.004	0.160	0.912
c^S	Shadow drive cleavage rate	0.098	0.012	0.080	0.099
$s_{c/g}$	Fitness cost per Cas9/gRNA allele	0.275	0.260	0.272	0.283
s_{NHEJ}	Fitness cost of single NHEJ allele	1.000	0.976	0.995	1.000

Supplementary Table 10 - "Linked" autosomal split-drive parameter estimates - *prosalpha2* (0). Parameters were estimated by running the log-likelihood (above) through a differential evolution MCMC algorithm. The maximum a posteriori estimate (M.A.P.) is the point that appears most often, and is the best point estimate for our parameters. Additionally, the mean and 95% credible intervals are provided. This drive is a *prosalpha2* sGD, where both parts are located on chromosome 3, close enough that cross-overs are negligible.

Parameters		Differential evolution MCMC parameter estimates			
**Label**	**Description**	**M.A.P.**	**2.5% quantile**	**Median**	**97.5% quantile**
c_F^H	Female cleavage rate	1.000	0.971	0.995	1.000
p_F^{HDR}	Female HDR rate	0.792	0.684	0.776	0.822
p_F^{NHEJ}	Female functional NHEJ rate	0.957	0.698	0.952	0.999
c_M^H	Male cleavage rate	0.998	0.982	0.996	1.000
p_M^{HDR}	Male HDR rate	0.807	0.801	0.814	0.872
p_M^{NHEJ}	Male functional NHEJ rate	0.990	0.751	0.961	0.999
c^S	Shadow drive cleavage rate	0.250	0.215	0.244	0.250
$s_{c/g}$	Fitness cost per Cas9/gRNA allele	0.000	0.000	0.000	0.001
s_{NHEJ}	Fitness cost of single NHEJ allele	0.006	0.001	0.021	0.367

Supplementary Table 11 - X-Linked split-drive parameter estimates - *rab11*. Parameters were estimated by running the log-likelihood (above) through a differential evolution MCMC algorithm. The maximum a posteriori estimate (M.A.P.) is the point that appears most often, and is the best point estimate for our parameters. Additionally, the mean and 95% credible intervals are provided. This is one of the two X-linked designs that begin in a true wild-type background.

Parameters		Differential evolution MCMC parameter estimates			
**Label**	**Description**	**M.A.P.**	**2.5% quantile**	**Median**	**97.5% quantile**
c_F^H	Female cleavage rate	1.000	0.991	0.998	1.000
p_F^{HDR}	Female HDR rate	1.000	0.991	0.998	1.000
p_F^{NHEJ}	Female functional NHEJ rate	0.173	0.016	0.408	0.965
c_M^H	Male cleavage rate	0.999	0.990	0.997	1.000
p_M^{HDR}	Male HDR rate	0.999	0.990	0.998	1.000
p_M^{NHEJ}	Male functional NHEJ rate	0.005	0.016	0.390	0.963
c^S	Shadow drive cleavage rate	0.098	0.055	0.092	0.100
$s_{c/g}$	Fitness cost per Cas9/gRNA allele	0.158	0.151	0.156	0.162
s_{NHEJ}	Fitness cost of single NHEJ allele	0.425	0.025	0.319	0.493

Supplementary Table 12 - X-linked split-drive parameter estimates – *prosalpha2* (1). Parameters were estimated by running the log-likelihood (above) through a differential evolution MCMC algorithm. The maximum a posteriori estimate (M.A.P.) is the point that appears most often, and is the best point estimate for our parameters. Additionally, the mean and 95% credible intervals are provided. This is the second of the two designs using X-linked Cas9 that begin in a true wild-type background.

Parameters		Differential evolution MCMC parameter estimates			
**Label**	**Description**	**M.A.P.**	**2.5% quantile**	**Median**	**97.5% quantile**
c_F^H	Female cleavage rate	1.000	0.994	0.999	1.000
p_F^{HDR}	Female HDR rate	1.000	0.991	0.998	1.000
p_F^{NHEJ}	Female functional NHEJ rate	0.601	0.027	0.517	0.978
c_M^H	Male cleavage rate	0.998	0.973	0.995	1.000
p_M^{HDR}	Male HDR rate	0.917	0.882	0.917	0.946
p_M^{NHEJ}	Male functional NHEJ rate	0.971	0.505	0.883	0.996
c^S	Shadow drive cleavage rate	0.059	0.003	0.051	0.098
$s_{c/g}$	Fitness cost per Cas9/gRNA allele	0.000	0.000	0.000	0.002
s_{NHEJ}	Fitness cost of single NHEJ allele	0.267	0.006	0.183	0.483

Supplementary Table 13 - X-linked split-drive parameter estimates – *prosalpha2* (2). Parameters were estimated by running the log-likelihood (above) through a differential evolution MCMC algorithm. The maximum a posteriori estimate (M.A.P.) is the point that appears most often, and is the best point estimate for our parameters. Additionally, the mean and 95% credible intervals are provided. This is the design performed in a 100% X-linked Cas9 background.

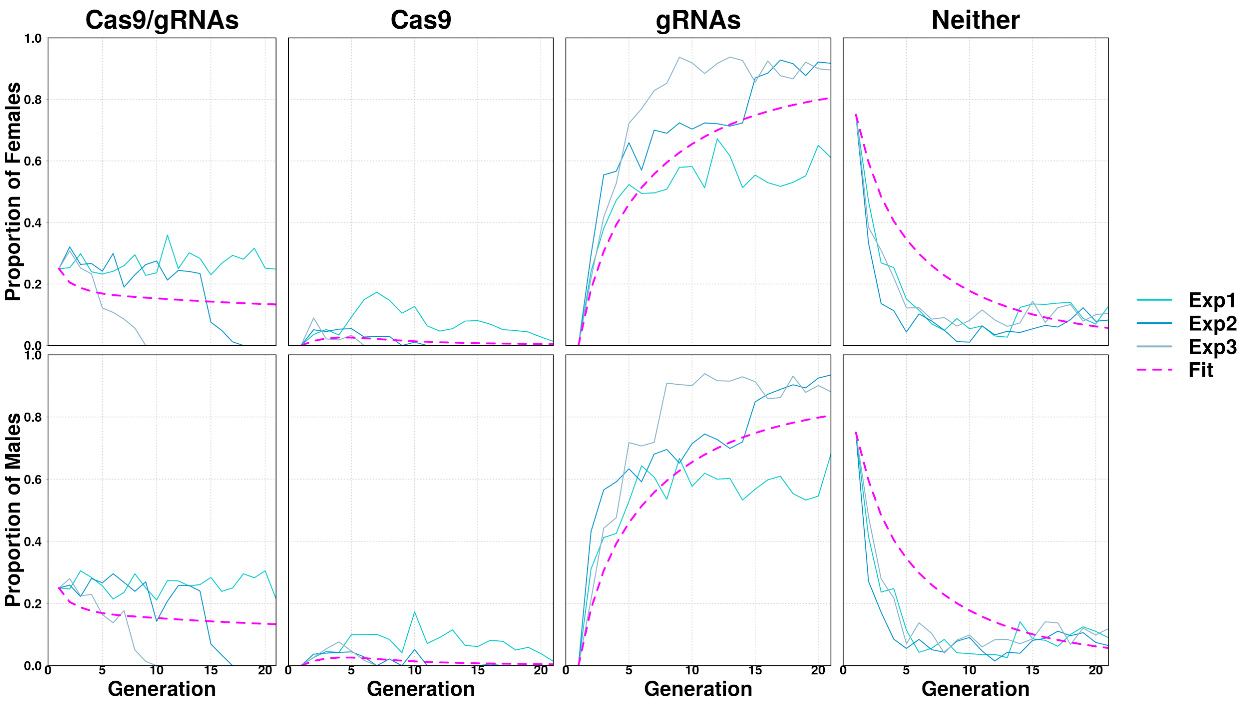

Supplementary Figure 6 - Autosomal cage trials and model fit - *rab5*. Observed and model-predicted population dynamics for the autosomal rab5 split-drive. The genotype-to-phenotype mapping is provided in Table S4 and the parameter estimates in Table S7. Model predictions use a deterministic version of the model, the same version used in the MCMC fitting procedure.

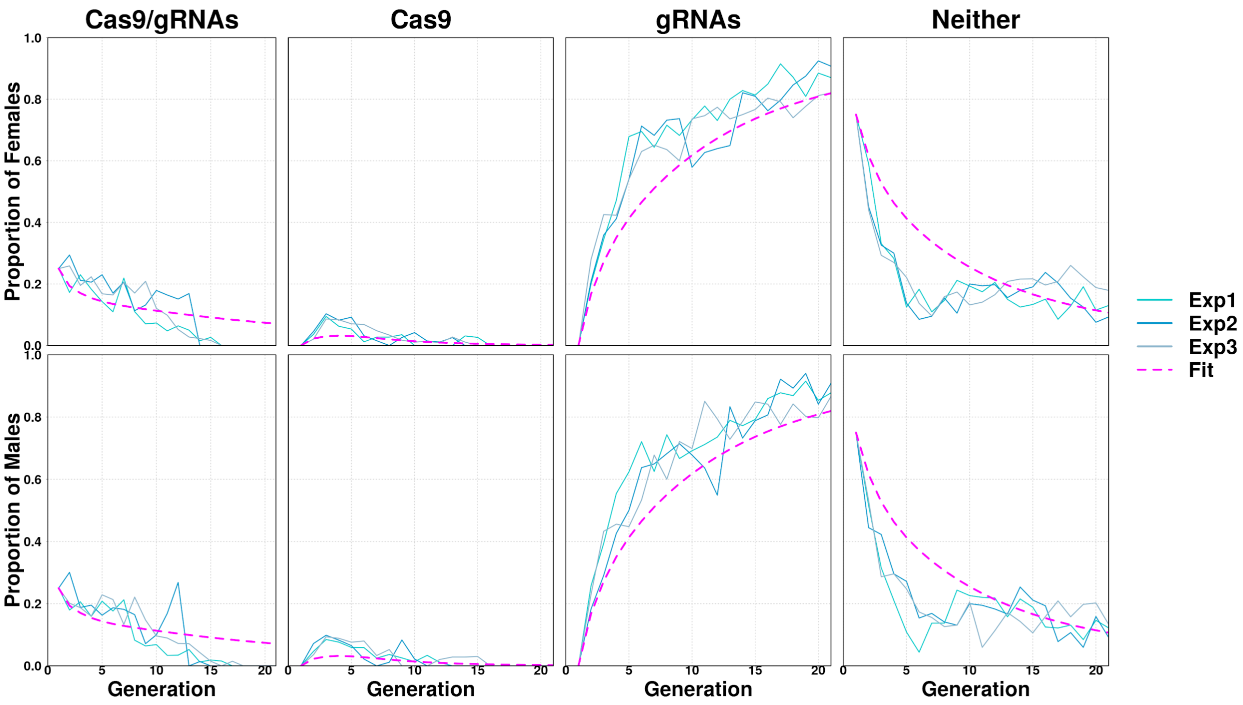

Supplementary Figure 7 - Autosomal cage trials and model fit - *spo11*. Observed and model-predicted population dynamics for the autosomal spo11 split-drive. The genotype-to-phenotype mapping is provided in Table S4 and the parameter estimates in Table S8. Model predictions use a deterministic version of the model, the same version used in the MCMC fitting procedure.

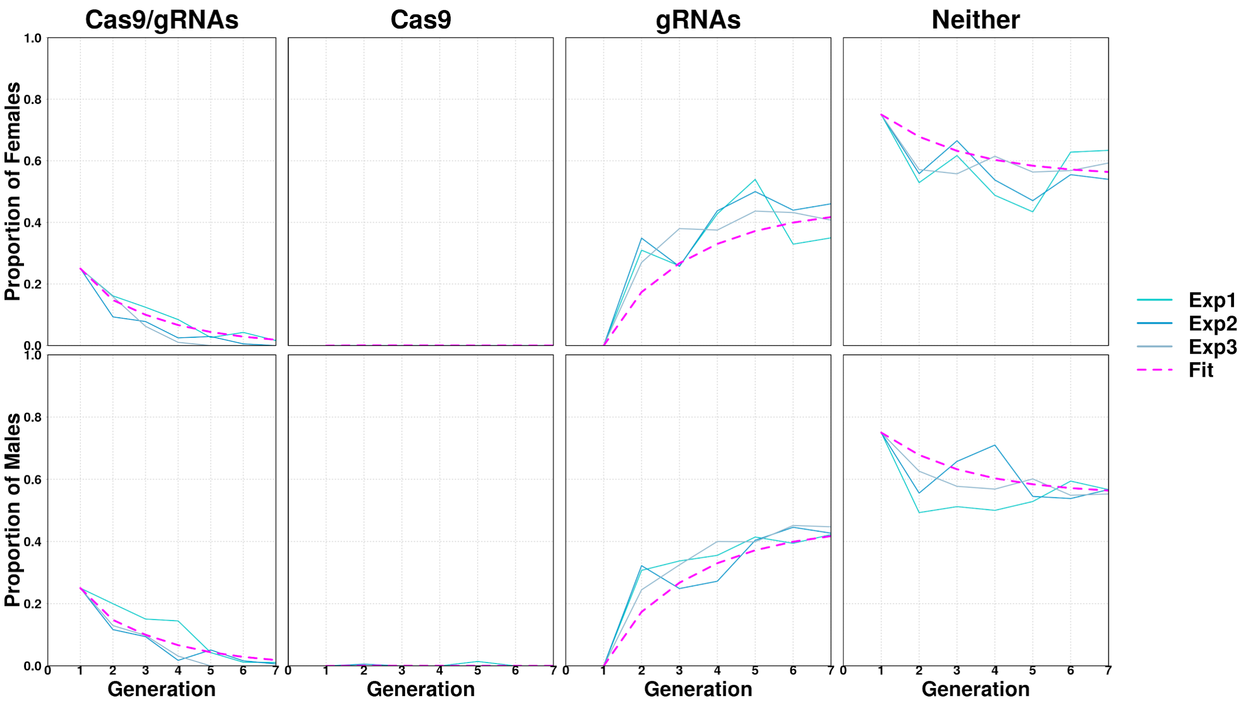

Supplementary Figure 8 - “Linked” autosomal cage trials and model fit – *prosalpha2* (0). Observed and model-predicted population dynamics for the autosomal *prosalpha2* (0) sGD. The genotype-to-phenotype mapping is provided in Table S5 and the parameter estimates in Table S9. Model predictions use a deterministic version of the model, the same version used in the MCMC fitting procedure.

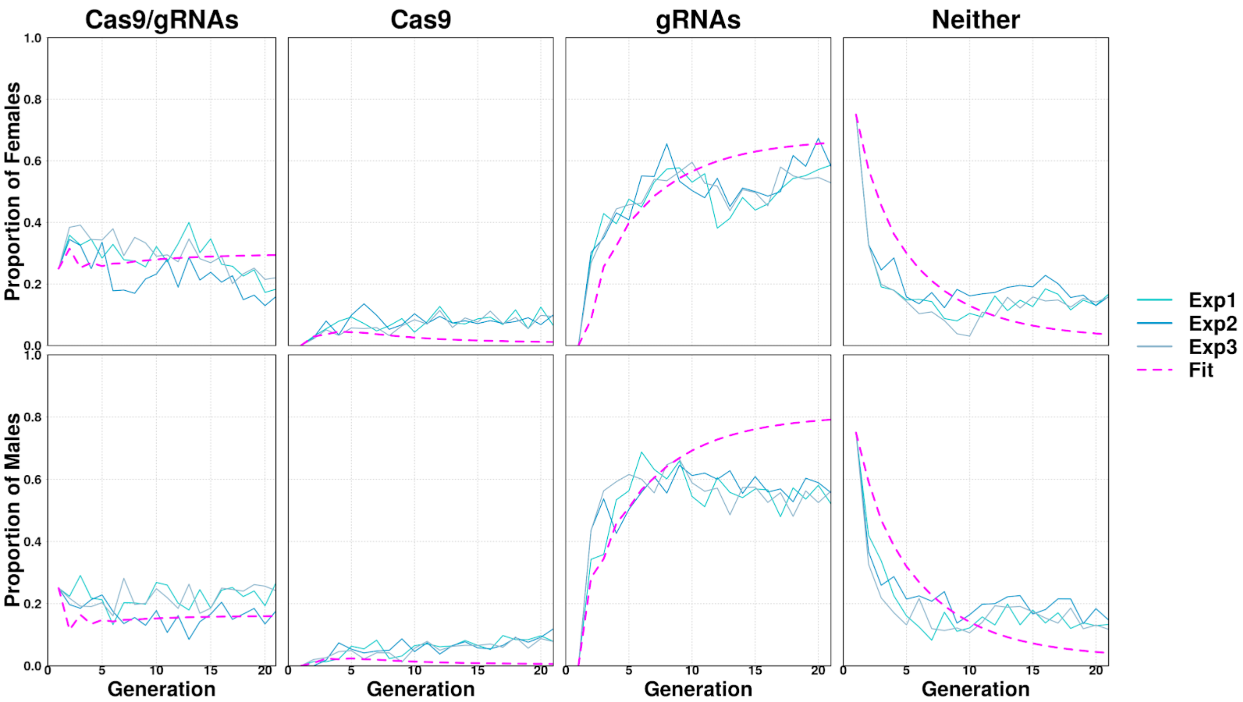

Supplementary Figure 9 - X-linked cage trials and model fit - *rab11*. Observed and model-predicted population dynamics for the X-linked Cas9; *rab11* sGD. The genotype-to-phenotype mapping is provided in Table S6 and the parameter estimates in Table S10. Model predictions use a deterministic version of the model, the same version used in the MCMC fitting procedure.

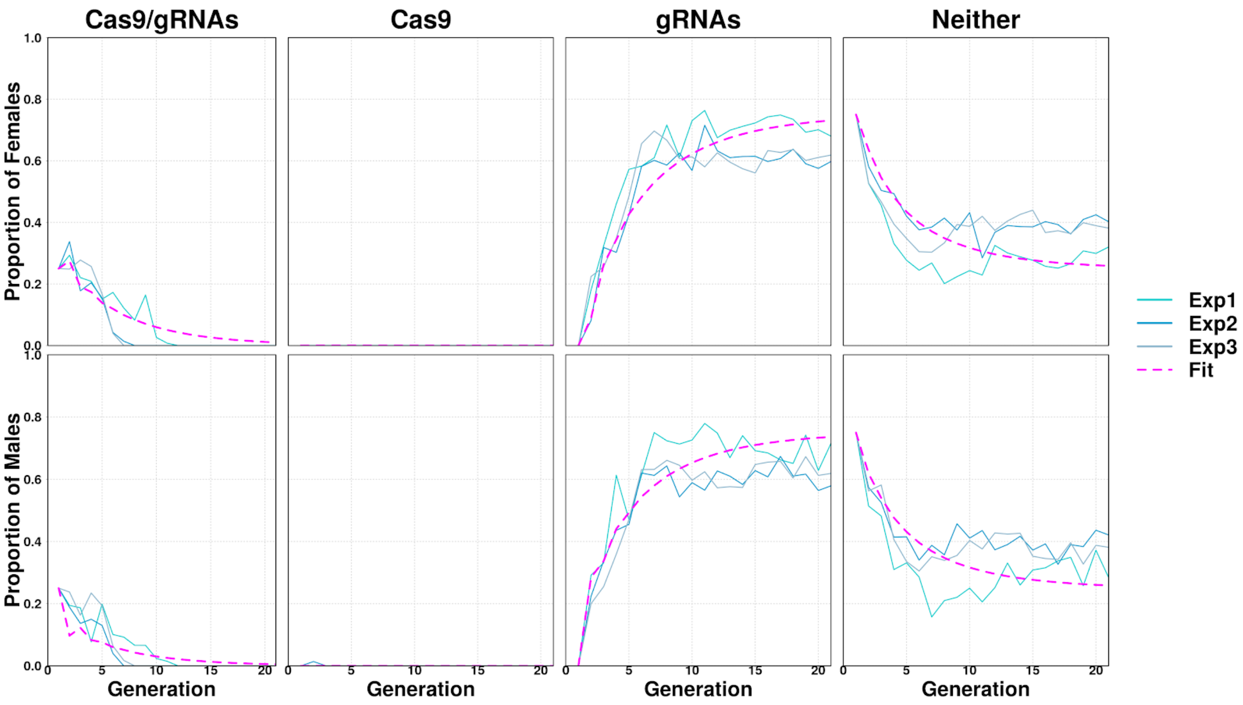

Supplementary Figure 10 - X-linked cage trials and model fit – *prosalpha2* (1). Observed and model-predicted population dynamics for the X-linked Cas9; *prosalpha2* sGD. The genotype-to-phenotype mapping is provided in Table S6 and the parameter estimates in Table S11. Model predictions use a deterministic version of the model, the same version used in the MCMC fitting procedure.

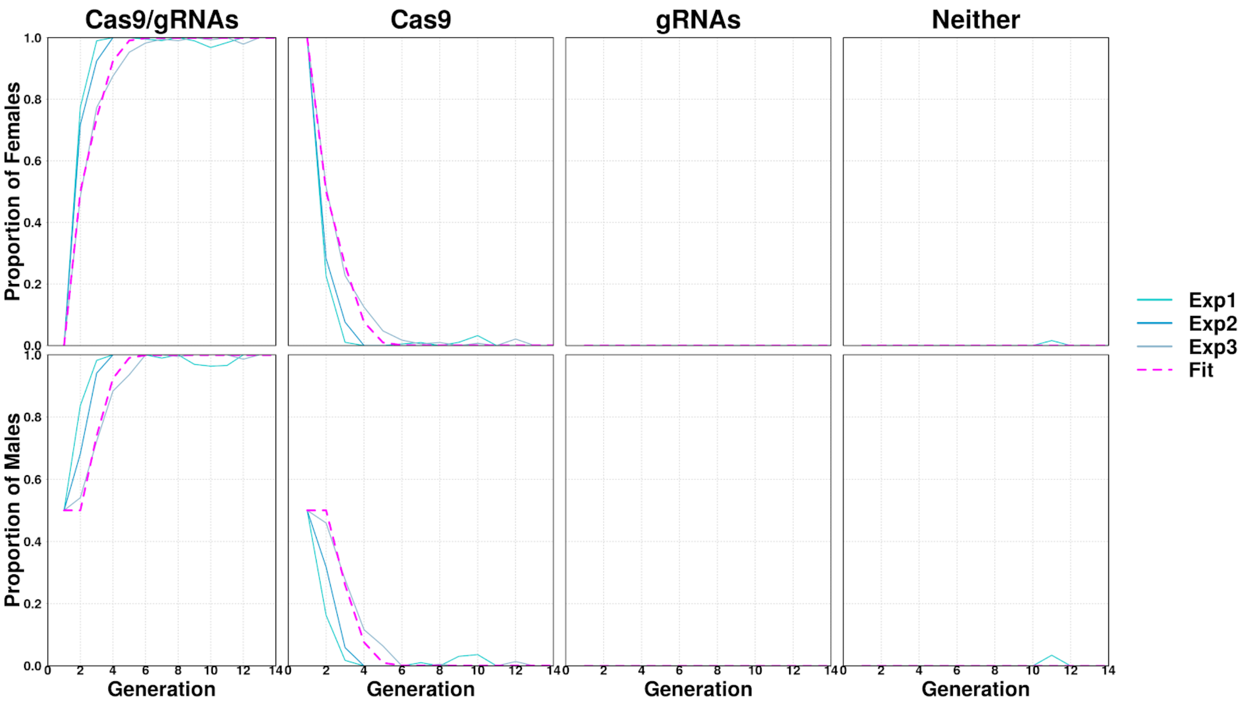

Supplementary Figure 11 - X-linked cage trials and model fit – *prosalpha2* (2). Observed and model-predicted population dynamics for the X-linked Cas9; *prosalpha2* sGD released into a 100% Cas9 background. The genotype-to-phenotype mapping is provided in Table S6 and the parameter estimates in Table S12. Model predictions use a deterministic version of the model, the same version used in the MCMC fitting procedure.
