# Supplementary material for: Inherently confinable split-drive systems in Drosophila
Source: Nat Commun. 2021 Mar 5;12:1480. doi: 10.1038/s41467-021-21771-7 (PMC7935863; doi:10.1038/s41467-021-21771-7)
Supplement: Supplementary file 2 — Description of Additonal Supplementary files [file 41467_2021_21771_MOESM2_ESM.docx]

**Title: Supplementary Data 1 – Phenotypic analysis of the F_2_ progeny from the single crosses (sGD+Cas9 source) performed and depicted in Figure 2.**

**Description:** Raw data counts for the F_2_ single cross progeny. Red marker (sGD), green marker (Cas9), both (sGD+Cas9) or no fluorescence (WT) were scored in order to track the sGD and Cas9 transgenes. Males and females were counted independently and merged to calculate the transgene inheritance rates. Data is divided in different tabs, separated by sGD and Cas9 source.

**Title: Supplementary Data 2 - Phenotypic analysis of the generational cage trials performed and depicted in Figure 4.**

**Description:** Raw data counts for the generational cage trials. Red marker (sGD), green marker (Cas9), both (sGD+Cas9) or no fluorescence (WT) were scored in order to track the dynamics of the sGD and Cas9 transgenes. At each generation, males and females were scored independently and merged to calculate the transgene % in each cage. Data is divided in different tabs, separated by sGD and Cas9 source.

**Title: Supplementary Data 3 – Raw data for Supplementary Figures.**

**Description:** Raw data counts for Supplementary Figures 2 and 3.

Supplementary Fig.2 consists of a different way of looking at the data shown in Figure 2. Hence, its tab structures the data the same way that it is presented, separated by sGD and independent of Cas9 source (shown in different colors).

Tabs for Supplementary Fig.3 show raw data counts for the F_3_ progeny needed for shadow drive experiments. Red marker (sGD), green marker (Cas9), both (sGD+Cas9) or no fluorescence (WT) were scored in order to track the sGD and Cas9 transgenes. Males and females were counted independently and merged to calculate the transgene inheritance rates. Data is divided in different tabs, separated by sGD
