# Supplementary material for: Inherently confinable split-drive systems in Drosophila
Source: Nat Commun. 2021 Mar 5;12:1480. doi: 10.1038/s41467-021-21771-7 (PMC7935863; doi:10.1038/s41467-021-21771-7)
Supplement: Supplementary file 7 — Reporting Summary [file 41467_2021_21771_MOESM7_ESM.pdf]

nature research

Corresponding author(s): Ethan Bier

Last updated by author(s): Jan 26, 2021

Reporting Summary

Nature Research wishes to improve the reproducibility of the work that we publish. This form provides structure for consistency and transparency in reporting. For further information on Nature Research policies, see our Editorial Policies and the Editorial Policy Checklist.

Statistics

For all statistical analyses, confirm that the following items are present in the figure legend, table legend, main text, or Methods section.

n/a	Confirmed	
☐	☒	The exact sample size (*n*) for each experimental group/condition, given as a discrete number and unit of measurement
☐	☒	A statement on whether measurements were taken from distinct samples or whether the same sample was measured repeatedly
☐	☒	The statistical test(s) used AND whether they are one- or two-sided<br>*Only common tests should be described solely by name; describe more complex techniques in the Methods section.*
☒	☐	A description of all covariates tested
☒	☐	A description of any assumptions or corrections, such as tests of normality and adjustment for multiple comparisons
☐	☒	A full description of the statistical parameters including central tendency (e.g. means) or other basic estimates (e.g. regression coefficient) AND variation (e.g. standard deviation) or associated estimates of uncertainty (e.g. confidence intervals)
☐	☒	For null hypothesis testing, the test statistic (e.g. *F*, *t*, *r*) with confidence intervals, effect sizes, degrees of freedom and *P* value noted<br>*Give P values as exact values whenever suitable.*
☐	☒	For Bayesian analysis, information on the choice of priors and Markov chain Monte Carlo settings
☒	☐	For hierarchical and complex designs, identification of the appropriate level for tests and full reporting of outcomes
☒	☐	Estimates of effect sizes (e.g. Cohen's *d*, Pearson's *r*), indicating how they were calculated

Our web collection on statistics for biologists contains articles on many of the points above.

Software and code

Policy information about availability of computer code

Data collection	No software was used for data collection.
Data analysis	Data statistical analyses were done using GraphPad's Prism 8 and potentially modified using Illustrator CS6. A version of MGDrivE (https://marshalllab.github.io/MGDrivE/) was used for simulation modeling.

For manuscripts utilizing custom algorithms or software that are central to the research but not yet described in published literature, software must be made available to editors and reviewers. We strongly encourage code deposition in a community repository (e.g. GitHub). See the Nature Research guidelines for submitting code & software for further information.

Data

Policy information about availability of data

All manuscripts must include a data availability statement. This statement should provide the following information, where applicable:

- Accession codes, unique identifiers, or web links for publicly available datasets
- A list of figures that have associated raw data
- A description of any restrictions on data availability

The sequence of all plasmid constructs generated in this paper has been deposited into the GenBank database after acceptance of the manuscript. Accession numbers are provided in the manuscript. Raw data, important primer sequences and mathematical modeling parameters are also be accessible as Supplementary information. The rest (cloning strategies and cloning primers) is available from the authors upon request.

Field-specific reporting

Please select the one below that is the best fit for your research. If you are not sure, read the appropriate sections before making your selection.

☒ Life sciences ☐ Behavioural & social sciences ☐ Ecological, evolutionary & environmental sciences

For a reference copy of the document with all sections, see nature.com/documents/nr-reporting-summary-flat.pdf

Life sciences study design

All studies must disclose on these points even when the disclosure is negative.

Sample size	For single pair-crosses (Fig 2), sample size was based on number of tested vials. We determined that 25+ independent crosses containing more than 30 progeny flies were enough to assure to avoid randomness or any unwanted biases to the result. For cage trials (Fig 4), we tested 3 cages for each condition to ensure proper reproducibility.
Data exclusions	No data were excluded from the analyses.
Replication	Reproducibility in single-pair crosses (Fig 2) was determined by N and n in each condition. In cage trials (Fig 4), it was determined looking at the variation among the 3 tested cages.
Randomization	Randomization was not relevant to the study. All experiments were performed using controlled genotypes and similar age individuals. Collection occurred 10 days after hatching.
Blinding	In cage trials where half of the population is used for seeding the next generation, we used a random half if both piles displayed similar phenotypic proportions. However, if that was not the case, the piles were altered to stay true to the total proportions of the cage to pass onto the next generation.

Reporting for specific materials, systems and methods

We require information from authors about some types of materials, experimental systems and methods used in many studies. Here, indicate whether each material, system or method listed is relevant to your study. If you are not sure if a list item applies to your research, read the appropriate section before selecting a response.

Materials & experimental systems

n/a	Involved in the study	
☒	☐	Antibodies
☒	☐	Eukaryotic cell lines
☒	☐	Palaeontology and archaeology
☐	☒	Animals and other organisms
☒	☐	Human research participants
☒	☐	Clinical data
☒	☐	Dual use research of concern

Methods

n/a	Involved in the study	
☒	☐	ChIP-seq
☒	☐	Flow cytometry
☒	☐	MRI-based neuroimaging

Animals and other organisms

Policy information about studies involving animals; ARRIVE guidelines recommended for reporting animal research

Laboratory animals	This study used male and female transgenic, balancer (II/III chromosome) and w1118 Drosophila melanogaster lines, from embryo to adult.
Wild animals	Study did not involve wild animals.
Field-collected samples	Study did not involve samples collected from the field.
Ethics oversight	All fly crosses were performed in glass vials in an ACL1 facility, in accordance with the Institutional Biosafety Committee-approved protocol from the University of California San Diego.

Note that full information on the approval of the study protocol must also be provided in the manuscript.
